# Mr. H. J. Paterson, F.R.C.S., with His Camera

**Published:** 1918-01-19

**Authors:** 


### MR. H. J. PATERSON, F.R.C.S., WITH HIS CAMERA.

### Some Remarkable Views.

ON Thursday, January 10, a lecture was delivered at the Home of the Royal Society of Medicine, under the auspices of the Royal British Nurses' Association, by the Medical Honorary Secretary of that body, Mr. Herbert J. Paterson, M.C., F.R.C.S. The lecture was illustrated by many slides.

The chair was occupied by the American Ambassador, Dr. W. H. Page, who said it was an admirable idea to hold the course of lectures which the Association had projected, and the subjects chosen were certainly important, especially under present conditions. Americans considered it a great privilege, even before they definitely entered the war, to send some medical succour to the armies in France; and, now that his country had taken sides, that had largely increased, and would increase still more. Recently 100,000 Red Cross nurses in training for Europe marched through New York, and many had now asked the privilege of being allowed to serve in Palestine.

Mr. Paterson racily described the voyage out, his impressions of the people and their customs, as well as the delights and inconveniences of travel there. He also portrayed distinguished American surgeons operating in their clinics, these including Dr. Finney, of Atlantic City; Dr. Bevan and Dr. John B. Murphy, of Chicago. Rochester, the locale of the world-famous Mayo clinic, he regarded as the home of American surgery. Here, as in all American hospitals, the anæsthetics are given by women nurses. After the diagnosis has been made and an operation decided upon, the patient is admitted to the Mayo Hospital. All the patients are private patients, and pay fees to the surgeons and to the hospitals for their accommodation. The Mayo Hospital resembles a London hospital, but here the resemblance ends, for in America there is no fettering and cramping lay control, which the surgeons there would not tolerate. The hospital at Rochester is managed by a committee of one, and he is a medical man. In this country the battle against lay control has still to be fought in the medical and nursing professions. Though the standard of surgery in Britain is to-day higher than ever, it no longer leads, for America enjoys freedom from lay control. British surgery has been cramped by lay boards; such boards favour large staffs, and these tend towards inefficiency. At Rochester there are five surgeons to nearly 400 beds; as the patients after operations are transferred to an annexe within a week or a fortnight, the volume of work approximates to that in a London hospital with 800 beds. The English system, instead of training one or two highly efficient men, tends to produce a number of mediocrities. It is inexplicable that in this country the expert is made subservient to the administrator; indeed in some instances the administrator seems to have been chosen in virtue of his ignorance of the work to be controlled. The British surgeon is handicapped by curtailment of opportunity, not by lack of ability.

In 1886 the first professional journal in the United States devoted to nursing was published, under the title *The Nightingale*. A criticism at the time said, "A magazine for nurses was uncalled for, improper, and capable of doing harm." Though the Royal British Nursing Association was founded in 1887 for the purpose of instituting a system of registration for nurses, and though for thirty years that profession has been trying to secure State registration, they have not yet secured it. Such registration has now been secured in America in at least forty-six States since 1903. For the backward state of affairs in this respect here the medical profession are not wholly free from blame, and the hampering effect of lay boards also has had much to do with it. But this condition of things must not be allowed to continue; the work of the nursing profession in the war has been so magnificent that common justice will compel Parliament to listen to the representations of the profession and to grant what is their just due. The training of American nurses is more systematic and more thorough than that of British nurses. Most of the large American training schools have a preparatory six months' course of instruction before the aspiring nurse enters the wards, but that practice probably prevails at not more than three London hospitals. Mr. Paterson, in conclusion, said he had now adopted the American system at his hospital, and had secured a trained nurse to help him in his operations, a course which he had never yet had cause to regret. He did not venture to introduce masks straight away, but now they also are included in the operating-theatre equipment. He paid a glowing tribute to the enthusiasm, charm, and hospitality of the American people.

Resolutions thanking Mr. Paterson for his lecture, and Dr. Page for presiding, were cordially passed; and the latter, in acknowledging the compliment to himself, said Americans had inherited to a certain degree British hospitality; they could not surpass it, and if any were able to visit his country they would find the men and women of the States quite glad to see them, and ready to extend to their visitors a hospitality which was both genuine and spontaneous.

The next lecture will be held in the rooms of the Medical Society on January 24, the subject being "Child Life."

*Note.*—See "Ierne's" article on "Lay Control," p. 336.

